# Strengthening resilience to emerging vector-borne diseases in Europe: lessons learnt from countries facing endemic transmission

**DOI:** 10.1016/j.lanepe.2025.101271

**Published:** 2025-04-04

**Authors:** Gina E.C. Charnley, Tilly Alcayna, Alex Almuedo-Riera, Christiana Antoniou, Athanase Badolo, Frederic Bartumeus, Laura-Lee Boodram, Rubén Bueno-Marí, Claudia Codeço, Flavio Codeço Coelho, Federico Costa, Horace Cox, Nabil Haddad, Nurulhusna Ab Hamid, Pattamaporn Kittayapong, Gülay Korukluoğlu, Antonios Michaelakis, Rafael Maciel-de-Freitas, Tomas Montalvo, Jose Muñoz, Silvia Sauleda Oliveras, John R.B. Palmer, Carlos Jesús Barboza Pizard, Guilherme S. Ribeiro, Rachel Lowe

**Affiliations:** a Barcelona Supercomputing Center (BSC), Barcelona, Spain; b School of Public Health, Imperial College London, London, United Kingdom; c London School of Hygiene & Tropical Medicine, London, United Kingdom; d Red Cross Red Crescent Climate Centre, The Hague, the Netherlands; e Barcelona Institute for Global Health (ISGlobal), Universitat de Barcelona, Barcelona, Spain; f International Health Department, Hospital Clínic de Barcelona, Barcelona, Spain; g Ministry of Health, Nicosia, Cyprus; h Laboratoire d’Entomologie Fondamentale et Appliquée, Université Joseph Ki-Zerbo, Ouagadougou, Burkina Faso; i Centre for Advanced Studies of Blanes (CEAB-CSIC), Girona, Spain; j Catalan Institution for Research & Advanced Studies (ICREA), Barcelona, Spain; k The Caribbean Public Health Agency, Port of Spain, Trinidad & Tobago; l European Vector Control Center of Excellence, Rentokil Initial, Madrid, Spain; m Parasites and Health Research Group, Department of Pharmacy, Pharmaceutical Technology and Parasitology, Faculty of Pharmacy, University of Valencia, Burjassot, València, Spain; n Programa de Computação Cientifica, Fiocruz, Rio de Janeiro, Brazil; o Fundação Getulio Vargas, Rio de Janeiro, Brazil; p Instituto de Saúde Coletiva, Universidade Federal da Bahia, Salvador, Brazil; q Medical Laboratory Sciences Program, Division of Health Professions, Faculty of Health Sciences, American University of Beirut, Beirut, Lebanon; r Institute for Medical Research, National Institutes of Health, Ministry of Health, Malaysia; s Center of Excellence for Vectors and Vector-Borne Diseases, Faculty of Science, Mahidol University, Nakhon Pathom, Thailand; t University of Health Sciences, Ankara Bilkent City Hospital, Türkiye; u Benaki Phytopathological Institute, Kifissia, Athens, Greece; v Bernhard Nocht Institute for Tropical Medicine, Hamburg, Germany; w Instituto Oswaldo Cruz, Fiocruz, Rio de Janeiro, Brazil; x Agència de Salut Pública de Barcelona, Barcelona, Spain; y CIBER Epidemiologia y Salud Publica (CIBERESP), Madrid, Spain; z Banc de Sang i Teixits, Barcelona, Spain; aa Universitat Pompeu Fabra, Barcelona, Spain; ab Ministerio de Salud Pública de Uruguay, Montevideo, Uruguay; ac Instituto Gonçalo Moniz, Fiocruz, Salvador, Brazil; ad Faculdade de Medicina da Bahia, Universidade Federal da Bahia, Salvador, Brazil

**Keywords:** Vector-borne diseases, Emerging infectious diseases, Climate change, One Health, Interventions, Vector control, Europe

## Abstract

Emerging vector-borne diseases (VBDs) are a major public health concern worldwide. Climate change, environmental degradation and globalisation have led to an expansion in the range of many vectors and an erosion of transmission barriers, increasing human exposure to new pathogens and the risk for emerging VBD outbreaks. Europe is potentially underprepared for the increasing threat of VBDs, due to attention and funding being diverted to other public health priorities. Proactive, rather than reactive, prevention and control approaches can greatly reduce the socio-economic toll of VBDs. Endemic countries globally have decades of experience in controlling VBDs, and Europe has much to learn from this knowledge. Here, we advocate for the expansion of transdisciplinary knowledge-sharing partnerships, to co-create proactive measures against VBDs. We present the experiences and expertise of our diverse international team and explore how an array of interventions can be applied and adapted to the European context.

**Search strategy and selection criteria**

To evaluate how best to integrate knowledge from endemic settings into European vector control for emerging pathogens, we first identified the current best practices and most recent literature on the subject. We solely used free text search terms (not Medical Subject Headings) to search Google Scholar and PubMed in June 2024, these terms included: “climate-sensitive infectious disease”, “arbovirus”, “Europe”, “vector”, “mosquito”, “tick”, “sandfly”, “Aedes”, “Crimean-Congo Haemorrhagic Fever”, “West Nile Virus”, “malaria”, “cutaneous leishmaniasis”, “control” and “measures”. We applied a filter to identify papers after 2020, to identify the most recent literature about vector management in Europe. We included any material which focussed on controlling emerging vectors and vector-borne diseases in Europe, including reviews, commentaries and viewpoints. We excluded any material which was not published in a peer-reviewed journal and was not specific to vector-borne diseases or diseases which we considered as an emerging threat in Europe.

## Introduction

Emerging vector-borne diseases (VBDs), such as cutaneous leishmaniasis and Crimean-Congo Haemorrhagic Fever (CCHF) are a major public health concern worldwide.^1^ Of particular concern are arboviruses, also known as arthropod-borne viruses, which are a diverse group of viruses including dengue, chikungunya, Zika, West Nile virus (WNV) and Oropouche virus. Arboviruses have seen a rapid global expansion in the last few decades, including expansion into areas of Europe. Limited treatment options or vaccines exist for arboviruses and they can result in serious forms of disease e.g., severe dengue and congenital Zika syndrome.^2^ Furthermore, there has been a recent uptick in locally-acquired malaria cases and some small outbreaks in Europe, after years of successful eradication.^3^ Changes to vector distribution, behaviour and density, including mosquitos, ticks and sandflies have been linked to an expansion of favourable climatic conditions under climate change. Additional factors contributing to this expansion include globalised travel and trade and changing land use and biodiversity altering hosts and ecosystems.^4^^,^^5^ A significant threat is the spread of the highly invasive vector, *Aedes albopictus*, and the re-introduction of *Aedes aegypti* into Europe, which has led to local outbreaks of dengue and chikungunya.^6^, ^7^, ^8^

**Fig. 1:**
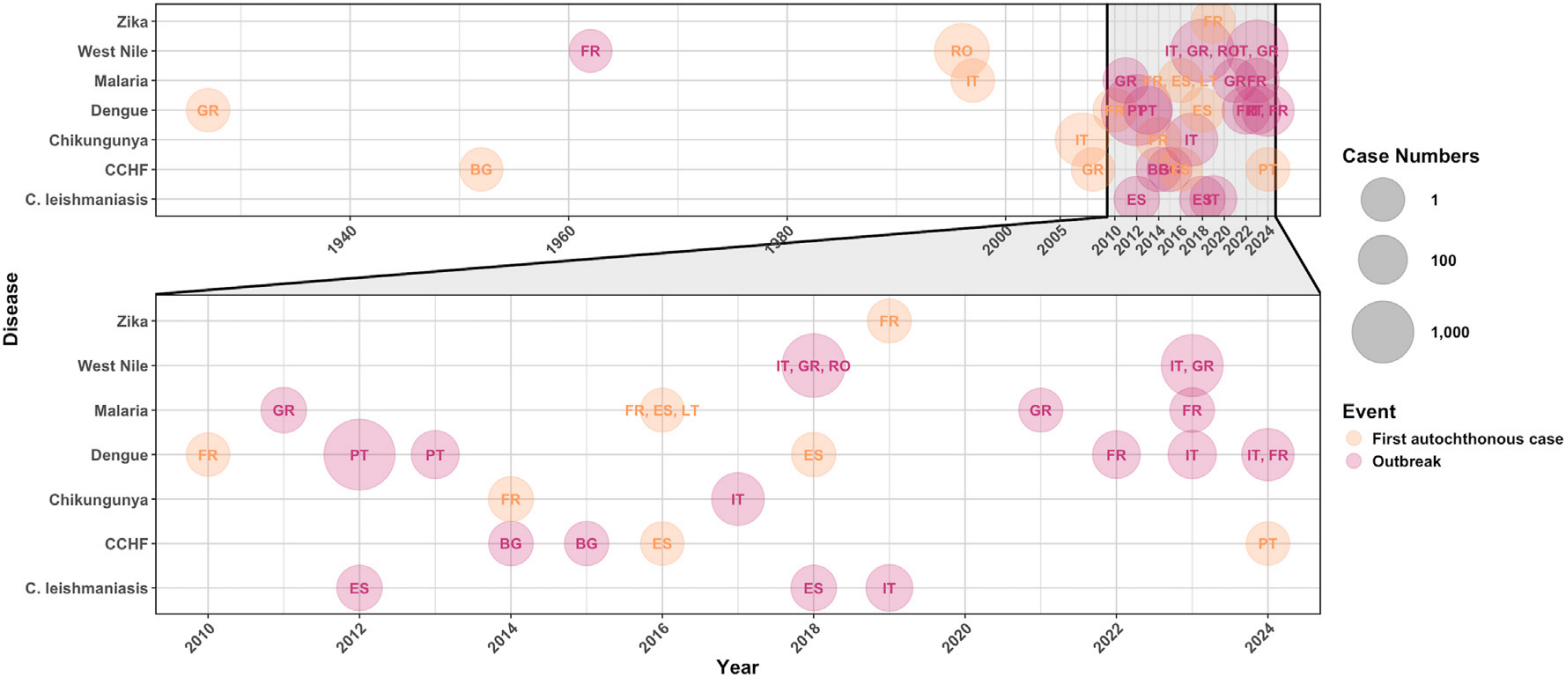
Timeline of first autochthonous cases and significant outbreaks (according to the European Centre for Disease Prevention and Control) of emerging VBDs in Europe since 1920, based on available information. Reports only include human cases confirmed via polymerase chain reaction, and not serological evidence. The labels represent the country/countries where the event was reported. The 2012 Portuguese (PT) outbreak of dengue was largely confined to the island of Madeira. The first autochthonous cases of malaria occurred since its eradication from Europe in the 1970s. C. leishmaniasis: Cutaneous leishmaniasis, CCHF: Crimean-Congo Haemorrhagic Fever. All European ISO2 codes are available in Table S1. Source: European Centre for Disease Prevention and Control.^9^

A convergence of ecologic, economic and social factors has resulted in a marked increase in VBD introductions and transmission in Europe over the last two decades (see Fig. 1).^2^ Some countries, especially those in the Mediterranean (e.g., southern France, Italy, Spain and Greece), have reported an expansion of several important vectors and/or autochthonous cases of emerging VBDs (see Figure S1 and Figure S2). The increase in VBD risk is of particular concern to European public health due to a largely immunologically naive population, and a lack of recent experience in dealing with major VBD threats, both of which could heighten morbidity and mortality.^10^ Historically, Europe has benefited from successful VBD eradication programmes, including those targeting malaria and plague, but these successes have also contributed to decisions to divert funding and attention away from VBD prevention towards other pressing public health issues. Yet, the global burden of mosquito-borne diseases related with invasive *Aedes* species is growing, with the costs far outpacing the amount spent on prevention—by about ten to one according to a recent study.^11^ The heavy toll can be expected to continue rising until more attention is given to proactive surveillance and control measures in place of the more reactive approach currently taken.

Despite the risk VBDs pose to public health, creating best practices for holistic vector control and disease prevention strategies remains a global challenge. Countries in sub-Saharan Africa, Asia and the Americas, are particularly affected by VBDs.^12^ Over time, the management of these diseases has led to extensive experience, creating a wealth of information, lessons learnt and expertise in the field of vector and disease prevention, surveillance and control strategies, which could provide valuable lessons for managing vectors and preparing for future VBD risks in Europe.

Here, we propose a toolkit of interventions to manage the rising risk of VBDs in Europe. We cover five broad themes and explore examples of their use in a range of settings, including two case studies. The themes include clinical case and risk management, vector management, adapting the built environment, data harmonisation and early warning systems, and community engagement. We explore the challenges and nuances around implementing these tools across diverse geographical and political settings along a spectrum of endemic and emerging disease transmission regimes. Europe is defined here according to the European Environmental Agency member and cooperating countries, and the United Kingdom (see Figure S3 and Table S1).^13^ The overarching goal is to help policymakers in Europe tailor disease preparedness and control strategies, which can be proactively implemented to increase resilience against the major emerging VBDs in Europe (see Table 1). We present how this could be achieved by promoting more transdisciplinary One Health partnerships, mobilising multiple sectors, disciplines and communities across society to work together at the human-animal-environment health interface.

**Table 1:** Major VBDs considered a threat to Europe in ascending order from first reported autochthonous cases in Europe.

Disease	Pathogen genus & organism type	Vectors	Non-human hosts	Current distribution & global burden	First autochthonous report in Europe
Malaria	*Plasmodium* parasite	*Anopheles* mosquitoes		Around 300–500 million malaria cases/year, mostly in Africa and the Guiana Shield	4000–3000 BC: Southern Europe
Cutaneous Leishmaniasis	*Leishmania* parasite	*Phlebotomus* sandflies	Dogs, rodents	Around 700,000 to 1 million reported cases/year in the Americas, the Mediterranean basin, the Middle East and central Asia	1756: Detailed clinical description of CL in Turkey
Crimean-Congo Haemorrhagic Fever (CCHF)	*Nairovirus* virus	*Hyalomma* ticks	Livestock (cattle, sheep & goats), wildlife	Around 10,000–15,000 cases per year in Africa, the Balkans, the Middle East and western and south-central Asia	1952: Confirmed in Bulgaria
West Nile Fever	*Flavivirus* virus	*Culex* mosquitoes	Birds, horses	From 2011 to 2022, 35,473 cases reported globally from North Africa, Europe, the Middle East, North America and Australia. In Europe, between March to October 2024, 19 countries reported 1436 autochthonous cases	1958: Serology in Albania 1996: Confirmed in Romania
Chikungunya	*Alphavirus* virus	*Aedes* mosquitoes		Since 2013, 3.7 million suspected and confirmed cases in the Americas. In Sept 2024, approximately 460,000 cases were reported worldwide	2007: Confirmed in Italy
Dengue	*Flavivirus* virus	*Aedes* mosquitoes		In 2023, 4.6 million cases across Asia, the Americas and Africa increasing to 7.6 million between January and April 2024. The Americas are currently experiencing the largest ever outbreak of dengue, between December 2024 and Februrary 2025 there have been over 600,000 cases	2010: Confirmed in France and Croatia
Zika	*Flavivirus* virus	*Aedes* mosquitoes		In 2024, 41,942 cases were reported in the Americas	2019: Confirmed in France
Oropouche Fever	*Orthobunyavirus* virus	*Culicoides* midges	Sloths, birds	In 2024, 11,634 cases were reported in the Americas	2024: No locally acquired cases but first travel-associated cases reported in Italy

The table includes the name of the disease, pathogen type, primary vectors and hosts, global burden of the disease in terms of cases and the first documented locally acquired case in Europe.^9^

## Clinical case & risk management

Clinical management of emerging VBDs poses many challenges, such as identifying best practices for testing and patient care that are sensitive to regional risk factors and demographics, along with difficulties distinguishing nonspecific symptoms and asymptomatic/mild clinical presentations. These issues are particularly prominent in emerging settings, as clinicians may not be aware of the risks and clinical signs of these diseases, and even if they are, correct testing and protocols may not be in place,^14^ particularly if the disease is not yet listed as notifiable by European Centre for Disease Prevention and Control (ECDC).^15^ Swift identification and management is essential in a clinical setting to prevent nosocomial transmission and implement infection preventive practices and control measures, such as access to polymerase chain reaction (PCR) and personal protective equipment (PPE).^16^ Professionals working in endemic settings have extensive knowledge in identifying and controlling VBDs in terms of identification, reporting, monitoring and managing emerging VBDs, at the clinical level.

Many VBDs of concern present as nonspecific febrile symptoms including fever, chills, myalgia and general malaise, particularly early in infection,^14^ leading to patients not seeking care and challenges to correct diagnosis. To bridge this gap and reduce the number of possible disease aetiologies, education is needed both for healthcare professionals and the general public. For example, ECDC publishes Weekly Threat Reports, enabling healthcare professionals to assess patient risk based on their geographic location in Europe.^17^ Furthermore, when the general public is more aware of risk factors they are more likely to be able to provide vital information to healthcare professionals, such as recent arthropod bites, travel to at-risk areas/countries, and potential exposure due to their occupation or recreational activities. For example, farmers, slaughterhouse workers, and veterinarians are considered at risk for CCHF, in areas where the disease is endemic in animals.^18^

Serological surveillance where possible can be used to monitor the population's current immunological status, helping to understand asymptomatic infections and the risk of outbreaks. Surveillance is important not only at the human level, but also for the intermediate/animal hosts, as well as the molecular biology of the vector.^19^ Monitoring studies in susceptible population are important to anticipate the risk and highlight potential exposures, to inform and prepare health workers to establish clinical case definitions, improve diagnosis and adopt case management protocols. For instance, in Lebanon, WNV is monitored through serological surveys of asymptomatic human populations living in areas ecologically suitable for transmission,^20^ along with chickens, horses and molecular tests of mosquitoes.^21^ Another method to assess disease burden and genetic diversity of VBDs at the population level is via blood bank testing, as WNV and dengue represent a serious threat to transfusion safety. Methods to ensure transfusion safety while keeping costs down and monitoring of emerging VBDs in healthy individuals are achieved via screening algorithms and testing with multiple assays, risk modelling, mini pool testing, quarantining blood components and reporting of emerging threats.^22^^,^^23^

In countries where arboviral risk is considered high, such as France and Spain, interventions are in place following clinical suspicion. Once healthcare professionals have identified possible aetiological agents, access to laboratory services to confirm current infections (such as PCR or blood smear) is essential to effectively identify, confirm and report emerging VBDs.^24^ The identification and management of cases associated with emerging VBDs should have a centralised recording system, to facilitate the creation of best practices for care and to allow for better collaboration between specialised hospitals and primary healthcare units. For example, in Türkiye, all information on patient care for confirmed and suspected cases is registered in their web-based system and can be accessed with a password by relevant government ministries, laboratories and healthcare professionals.

A vital component to any effective infectious disease management programme is adaptability. Systems in place need to be evaluated on a regular basis, incorporating feedback from those involved in the control and management of the disease at all levels and with a One Health approach in mind. Systems should be aware of and prepared for the effects of sudden unforeseen events, such as pandemics and sudden-onset conflicts. A combination of the strategies outlined here can be used to manage new threats and sudden shocks and crises. For example, a surge in leishmaniasis cases in 2013–2014 following the onset of the Syrian Civil War was recorded in Lebanon, predominantly amongst the large number of displaced Syrian refugees. Lebanese health authorities responded by implementing a comprehensive strategy which ramped up testing and access to care at health centres, provided education and awareness raising^25^ and targeted vector control of previously identified at-risk zones to control the outbreak.^26^

## Vector management

Mechanical and environmental approaches to controlling vectors have been used for millennia. For example, the use of some form of bed nets was reported in Ancient Egypt during the 5th Century BC and continues to be an effective strategy to reduce human exposure to mosquitoes.^1^ Drainage schemes were used in Greece as early as the 6th Century BC, and reports of other types of environmental controls can be seen throughout the historical record, for example, game destruction and bush clearing for tsetse control in the late 19th century in Sub-Saharan Africa.^1^ Furthermore, general improvements to basic services and poverty elimination have often coincided with a reduction in VBDs.^27^ The development of insecticides during the early 20th century led to a major shift in emphasis toward chemical control, particularly after the discovery of DDT in the early 1940s.^1^ Chemical control was used successfully against a range of VBDs, including typhus (lice), malaria (*Anopheles* mosquitoes), trypanosomiasis (tsetse flies), and leishmaniasis (sandflies).^1^ Chemical control, however, led to environmental damage, while also becoming decreasingly effective as over-reliance on insecticides drove resistance in vector populations.^1^

Today, some of the historical examples of successful chemical and environmental vector control measures may be deemed unsuitable for ethical and environmental reasons, and some are prohibited in European Union Member States.^28^ Examples include widespread culling of wildlife, drainage and clearance of ecosystems and liberal use of chemical insecticides, e.g., via aerial spraying.^1^ A rise in insecticide resistance and lack of new insecticides in development^29^ has created a need for innovative and integrated vector management. Furthermore, some diseases such as CCHF lack preventative measures, approved vaccines or effective treatments, making vector control and exposure prevention the main methods for disease control.^30^

Integrated pest management is key to effective vector control. Vectors sit within ecosystems, and their population dynamics depend on a wide range of factors, including land use and biodiversity changes.^28^ Some of these factors are being exploited today as a means of biological vector control, such as the use of water beetles and larvivorous fish as natural predators for mosquito larvae.^1^^,^^31^ Another important method is the sterile insect technique (SIT) which was developed in the 1930s and 40s as a way of controlling vectors without the use of chemicals. It involves releasing a large number of sterilised (via radiation) males of a target insect species into the environment, with the aim they will displace their non-sterile counterparts, reducing the overall population size.^32^ The classical SIT can also be used in combination with the incompatible insect technique (IIT), in which mosquitoes are infected with a strain of *Wolbachia*, a maternally inherited endosymbiotic bacteria that generates cytoplasmic incompatibility.^33^, ^34^ The radiation levels used in this approach are lower than those in classical SIT, yet the quality of males remains consistently high, ensuring their competitiveness regardless of the irradiation method (*Wolbachia* or classical SIT).^35^ Globally, SIT has proved to be an effective tool in integrated pest management for many Diptera insects, and was successful in Greece when used in combination with community engagement.^1^^,^^36^^,^^37^ Initial studies of IIT-SIT with *Wolbachia*-infected *Aedes* mosquitoes have shown promising results in the field in Southeast Asia^33^^,^^34^ and in small-scale field trials in Italy and laboratory tests in Spain,^38^^,^^39^ potentially being a useful tool for controlling *Ae. albopictus* in Europe in the future.

Like chemical and environmental vector control techniques, SIT and IIT can be fraught with logistical, legal, ethical, and environmental challenges, as well as poor community acceptance. Furthermore, SIT and IIT techniques present a number of unknown parameters that require further study. Laws vary across regions and countries; therefore, implementing vector control internationally cannot always be uniform. Within Europe, EU and non-EU countries (see Table S1 and Figure S3) will face different legal challenges in implementing vector control at a large scale. In the EU, *Wolbachia* is regulated as a biocide rather than a genetic modification, which may enable broader testing. At the global level, the World Health Organization (WHO) have already provided guidelines for safely implementing classical SIT.^40^

In order to ensure targeted and effective deployment of vector control, active surveillance of the vector is needed at different levels, to understand priority areas and detect any introduction events.^41^^,^^42^ Surveillance can be achieved via a variety of trapping methods and citizen science strategies.^43^^,^^44^ Some commonly used traps are CDC light traps, mosquito-oviposition traps and BG-Sentinel traps. Sampling bias is an issue with both trapping and citizen science, including trap placement effects, variable attraction to traps based on species and sex, and systematic differences in the places and times of citizen scientists’ reporting. Monitoring traps can be very resource intensive, which is considered one of the advantages to citizen science approaches. However, this comes with drawbacks related to uncertainty and credibility.^45^^,^^46^ Smart traps are being developed with sensors that use artificial intelligence to automatically classify vectors, to reduce the resources needed for identification.^43^^,^^47^ Trapping surveys provide vital information for regional and national surveillance programs, such as the surveillance in the Emilia-Romagna region of Italy, considered a WNV hotspot.^48^ At the regional level, the European Network for Medical and Veterinary Entomology (VectorNet) currently provides a centralised system for vector surveillance reporting.^49^ Europe should aim to continually improve the quality and the scale of its vector distribution data and maps as an essential tool in the control of VBDs.^37^

## Adapting the built environment

Urban settings have a distinct environment from rural areas, creating challenges and opportunities for different vectors. Differences include urban micro-climates due to phenomena such as the urban heat island effect, differing population densities and dynamics, more surfaces and containers that allow for water accumulation creating vector breeding sites and artificial lighting that can impact vector behaviour.^50^ Furthermore, city improvements and climate change adaptation measures like the expansion of urban green and blue spaces can create challenges for vector control.^51^ In response to urban outbreaks and the ability of some vectors to become highly adapted to the urban environment (e.g., *Ae. albopictus* and *Culex pipiens*), public health agencies are innovating vector control measures to reduce the opportunities for VBD outbreaks, via modifications to infrastructure, amenities and maintenance practices at both the public- and household-level.^52^

Infrastructure, amenities and maintenance modifications and practices can provide economical and long-term vector control by reducing vector breeding sites, potentially reducing the need for chemical control or other forms of control.^52^ For example, in Malaysia, frequent clearing of gutters is encouraged to reduce the accumulation of standing water.^38^ The WHO provides guidelines for modifications to create vector-resilient urban environments. At the household-level, WHO recommends interventions including having screens and seals on windows and doors, reducing wastewater and carefully managing stored water and waste.^53^ However, challenges arise in engaging and convincing individuals to remove breeding sites and reduce vector exposure on private properties.

At the public-level, WHO recommends actions to reduce the urban heat island effect and increase community knowledge and training.^54^ An example of a successful urban infrastructure modification at the public level is the modification of storm drains in Salvador, Brazil, after they were confirmed as resting and breeding sites for *Culex* and *Aedes* mosquitoes. The intervention involves placing concrete at the bottom of the drain to elevate the base on a gradient towards the outflow tube, improving drainage, preventing water accumulation and reducing mosquito activity.^54^, ^55^ It is important to conduct small-scale interventions in each area, ensuring stakeholder and community engagement, before wide-scale implementation. Local urban architecture, population behaviours and customs should be considered, and feasibility studies conducted before making larger financial investments.^56^

## Data harmonisation and decision-support tools

Collecting data on vectors, hosts and human disease is vital for VBD control, while the availability and accessibility of these data to researchers and decision-makers is important for quantifying risks and designing effective solutions. However, data needs to be harmonised for effective use in infectious disease decision-support modelling tools.^57^ Harmonisation allows for seamless linking of health data to climatic and environmental datasets, along with key socio-economic risk factors at common space-time scales. Robust statistical methodologies can then provide insight into early triggers and signals for VBD outbreaks, producing epidemiological alerts which can be used as a decision-support tool for timely action.^58^ For example, optimal temperature and humidity conditions facilitate dengue outbreaks in Singapore and extreme drought followed by wet conditions were predictive of dengue risk in Brazil.^59^^,^^60^ Furthermore, in Europe, spring temperatures have shown to be an early warning driver for WNV.^61^

An example of a decision support modelling tool is ‘Infodengue’, a nowcasting system for dengue, Zika and chikungunya, launched in 2015 in Brazil. The system was co-developed by researchers and state surveillance teams and integrates epidemiological, climate and social media data to provide real-time information on the transmission of dengue, communicating risk via a colour-coded classification system. Efforts are underway to expand the nowcasting system into an early warning system (EWS), by extending the predictive lead-time using tailored seasonal climate forecasts within a dengue early warning model framework.^62^^,^^63^

European agencies and institutions can learn from nowcasting systems, like Infodengue, and EWS, to co-create tailored tools for European emergence and outbreak risks. Transdisciplinary collaborations between public health agencies, veterinarians, entomologists, climate scientists, epidemiologists and ecologists are essential to co-create modelling tools to predict the risk and emergence of VBDs, as many emerging VBDs have complex transmission pathways, with multiple risk factors and hosts. To facilitate this One Health approach to knowledge transfer, data sharing and surveillance protocols should be harmonised as much as possible and unified among sources for resource allocation.^64^ Surveys to collect data for EWS should be large-scale and multi-pathogen, which offer cost benefits and better characterisation of transmission and immunology within the population e.g., more widespread surveillance, even in times considered “out-of-season”.

Early warning and nowcasting tools should aim to be methodologically flexible as many VBD risks are emerging in Europe and tools are likely to be improved as new data and knowledge become available. As stated above, many VBDs have nonspecific febrile symptoms, and can be difficult to diagnose. Time from symptom onset to diagnosis can be lengthy for many VBDs, even in endemic settings. In Europe, this delay is likely to be longer while education, awareness and testing are scaled up. For example, substantive reporting delays occurred during the 2017 chikungunya outbreak in Italy.^65^ These delays create issues when developing and updating EWS, and result in inaccurate or out-of-date disease risk alerts. To address the issues of testing delays in Europe, syndromic surveillance, and the monitoring of reported suspected cases may be a solution. This was found to be effective in Brazil to increase the sensitivity and timeliness of the alerts issued by Infodengue.^60^^,^^62^

Co-creation with decision-makers has been key to the success of Infodengue and other disease EWS for the Caribbean.^63^^,^^66^ EWS can serve as an educational and awareness-raising tool for healthcare professionals and the public, via their development, outbreak alerts and actionable contingency plans. Co-creation of EWS involving multiple stakeholders ensures that the output is easily interpretable by a range of end-users and is designed to trigger action plans. Open communication and partner meetings are needed to boost the system's adoption and continue to ensure that the system is appropriate for the desired end user. Co-creation approaches have been adopted by groups working on EWS in Europe (e.g., IDAlert,^67^ CLIMOS^68^), Asia (e.g., E4Warning^69^) and Latin America and the Caribbean (e.g., ENDCast^70^).

Looking beyond the sub-seasonal to seasonal timescales of many EWS, co-creation approaches have been used in indicator development for key epidemiological risks at global and regional scales. The Lancet Countdown^71^ is an international research collaboration monitoring climate and health. The consortium has developed a number of key infectious disease indicators, including arboviruses and malaria, tracking these indicators annually to understand the threats posed to global population health now and in the future. Global indicators provide a foundation for adaptation for regional use and an example of how international knowledge can be used in emerging settings such as Europe. The Lancet Countdown Europe^10^ and the project IDAlert^67^ have tailored many indicators used in the global Lancet report to a European setting, and in line with Europe-specific risks and needs. Further modelling studies can be used to improve the predictive performance of the tailored indicators, such as the multi-model framework (thresholds-based models, machines learning approaches & mechanistic transmission models) used in IDAlert,^67^ and scenario-based modelling to test the impacts of further global change (changes to the climate, land use, biodiversity and travel)^5^^,^^8^, ^72^ and interventions.^73^^,^^74^ Modelling challenges and sprints can be a useful way of co-creating robust and multi-model ensembles for prediction, such as those carried out by Infodengue in 2024 and by the US Center for Disease Control and Prevention in 2020 for WNV.^75^ Similar challenges could be of great use in Europe in bringing together epidemiological modelling expertise and developing EWS.

## Community engagement

Community engagement is a process of relationship building that empowers communities in decision-making, planning, and delivery of services and interventions and is a cornerstone to achieving positive health impacts and outcomes at the community level. Community engagement encompasses a variety of strategies and degrees of involvement of communities,^76^ and should consider geography, socio-economic status, educational attainment and gender to effectively influence the practice of appropriate surveillance and vector control.^77^

Knowledge, Attitudes and Practice (KAP) surveys provide important information on community-level awareness of risks, the effectiveness of interventions and community acceptance of vector control measures.^78^, ^79^, ^80^ In Europe, KAP surveys have been used in the context of vector and disease risk management, to identify what prevention measures are being taken, where community participation is most needed, what kind of behavioural changes are required, and what additional resources are needed to support change through public health education campaigns.^81^^,^^82^ For example, in Greece, it was found that while education campaigns are necessary, they cannot solely rely on information dissemination and need direct involvement from scientific staff and community leaders, especially in urban areas, to reduce breeding sites and control the spread of VBDs.^82^^,^^83^ Furthermore, education campaigns should be co-designed with communities to ensure uptake, reach and effectiveness.^36^ The monitoring and evaluation frameworks for each campaign must align with the findings of the KAP study and be structured such that short-, medium- and long-term outcomes are carefully tracked, and fed back into the campaign to enhance long-term effectiveness.

Community-based surveillance (CBS) is another form of community engagement that involves using community knowledge to identify and report health events, enhancing education and participation.^84^ CBS has been used globally in early detection of outbreaks and long-term surveillance for malaria in Guatemala^85^ and widely in humanitarian settings where human resources may be low.^36^ Previous interventions have included training individuals in rural communities to test (via blood smear) and identify malaria cases. Data collection can be active or passive, and CBS and other forms of citizen science for VBDs can provide scalable systems that can capture information at the highly localised level and aggregate these data over larger scales to understand incidence, distribution and patterns of vectors and diseases.^86^^,^^87^ Citizen science can enhance public awareness and engage the public in management and surveillance practices. Such forms of community engagement could be highly relevant in the European context where mobile phone ownership, use and coverage are high but may need to overcome the challenges of open innovation in the private sector.^88^^,^^89^

CBS and KAP surveys are not only used to implement household- and individual-level urban infrastructure changes and roll out large-scale vector management techniques such as *Wolbachia*.^54^^,^^83^ They can be used to create community-based interventions (CBI) tailored to local priorities. For example, following awareness workshops, community groups in Brazil developed “Boot Out”, an intervention focussing on eliminating municipal waste from public and private areas in collaboration with urban cleaning services. CBI can provide low-cost initiatives and unite communities to address health issues based on shared perceptions and scientific evidence.^90^ For example, community leaders in Brazil have assisted in out-of-pocket interventions to modify the structure of 52 storm drains located on streets serving private properties to prevent water accumulation.^54^ However, implementing these strategies in different countries can present challenges in relation to the law around private property access.

**Case Study 1: Barcelona, Spain**

Barcelona is a vibrant city of 1.7 million residents, divided into ten districts, with a total area of 10,137 ha. As the capital of the Catalonia region in northeastern Spain, it enjoys a distinctive setting on the Mediterranean coast, bordered by the Collserola mountains. This combination of coastal and mountainous geography creates a warm, humid climate with relatively low annual rainfall, characteristic of its Mediterranean climate. The city is an important hub not only for Spain, but for the whole Mediterranean region, due to its large air and sea ports.^91^ The combination of its climate and high throughput makes Barcelona a high-risk area for vector introduction, establishment and VBD outbreaks. Due to the VBD risks that Barcelona faces there have been multiple efforts and co-designed implementation strategies by scientists, government departments and citizens, to reduce these threats in Barcelona, relating to some of the toolkit themes outlined here:

1. Clinical case and risk management–CCHF is a viral disease with high morbidity and mortality, transmitted to humans primarily through bites of infected *Hyalomma* ticks or contact with infected blood or tissues.^16^ A retrospective study revealed the first case of CCHF in central Spain (Ávila, Castile-León) in 2013 and there have been 17 cases detected since but none in Catalonia, despite the presence of the vector and confirmed virus circulation in wildlife.^92^ Barcelona's national reference centre for managing imported tropical diseases, one of Spain's High-Level Isolation and Treatment Units, is coordinating the surveillance and response efforts for CCHF. Surveillance systems for humans, animals, and ticks could be combined and strengthened to support early detection and monitor virus clades, as these can influence clinical manifestations.^30^ Rapid laboratory diagnosis of CCHF infection is crucial and is being optimised via standardised case definitions and adequate laboratory capacity for molecular diagnostics, to ensure early recognition, isolation and supportive treatment. Treatment and reference centres provide specialised healthcare worker training and ensure safe working environments for disease management, operating 24/7 for continuous monitoring and rapid response.
2. Vector management–Spain has established populations of several vector mosquito species of the *Culex* and *Aedes* genera. Understanding the distribution of these vectors and monitoring the introduction of new species and their capacity for transmitting VBDs is vital for public health. In Barcelona, this is being carried out with a combination of smart traps (traps using automated AI-based mosquito classification), citizen science and traditional surveillance. Traditional mosquito surveillance in Barcelona has mainly consisted of the continuous surveillance of larval breeding sites in public spaces. This has been augmented at times with the use of adult mosquito traps and smart traps. One limitation of these approaches is their geographic constraints: they are largely confined to public spaces such as fire stations and other protected areas on local government property, to prevent theft or vandalization. Based on advice from colleagues in Malaysia who have deployed ovitraps in parks by hiding them in shrubs, ovitrap surveillance was used in certain Barcelona parks during the 2024 mosquito season. Additional spatial coverage has been gained through the “Mosquito Alert” citizen science system.^93^ Mosquito Alert enables users to submit photographs of mosquitoes they encounter, which are then reviewed by entomologists to identify mosquito species. The information is made available in near-real-time to end users. Early collaboration in its development included partners in Hong Kong, Italy, and the US, and the system is now used throughout Europe. More recently there has been work on implementing Mosquito Alert in various countries in Africa as well as in Bangladesh, Brazil, and Vietnam.
3. Adapting the built environment–After learning from the experiences of partners in Brazil, the Agència de Salut Pública de Barcelona and Barcelona Cicle de l’Aigua (BCASA) have coordinated an operation to test the feasibility of storm drain modification in Barcelona. When using the same modifications as in Brazil, Barcelona saw a significant reduction in *Aedes* and *Culex* activity in the modified drains.^52^^,^^56^ Further testing is now ongoing to determine the best approaches for storm drain modification and other breeding site removal city-wide. Sand sewer storm drains and ornamental fountains have been identified as primary vector mosquito breeding sites in public spaces. Sand sewer storm drains accumulate standing water because of their design, which was intended to prevent the sewer system from being clogged with sand entering from parks and streets. However, BCASA has concluded that this safeguard is no longer needed because upgrades to the rest of the system avoid clogging. Learning from partners in Brazil, the modification or elimination of these drains is being expanded to remove more vector breeding sites. These modifications have been consolidated in the city's water management policy, so that each year the most problematic structures are modified to gradually reduce the breeding sites city-wide.
4. Data harmonisation and decision-support tools–Barcelona's national reference centre publishes and shares data via monthly updates, which can contribute to advancing knowledge and improving future treatments.^94^ Plans are underway to share this data with key stakeholders involved in outbreak response. Integrating this information into local and regional emergency response services, referral and non-referral health centres, reference laboratories, and both human and veterinary health sectors could enhance transnational coordination. These efforts could be further improved by integrating decision-support tools and data harmonisation frameworks, facilitating real-time information exchange not only within Spain but also across Europe. A multidisciplinary One Health approach could be implemented, integrating knowledge from partners in CCHF-endemic countries to include mapping endemic areas and identifying potential expansion zones. Additionally, establishing a network of experts from different disciplines, both from endemic and non-endemic countries, could provide a platform for local response with highly specialised knowledge.
5. Community engagement–KAP surveys are a strategy used throughout Barcelona for the last three years to ensure public health education campaigns and are considered an important tool for identifying inequalities and vulnerable groups in the city, to act where communities are most in need. Additionally, educational workshops on reducing vector breeding sites have been held since 2016 for children aged 13–17 years old as a strategy of citizen involvement and collaboration and to help administrations in public areas. In the response to CCHF, engagement with at-risk groups (e.g., farmers and veterinarians) is recommended to help improve tick management, public health interventions, and awareness-raising activities.

**Case Study 2: Emergence of *Aedes aegypti***

Following the introduction and rapid expansion of *Ae. albopictus* in Europe, concerns have arisen that similar events could occur with other vectors capable of transmitting arboviruses.^6^ One vector of concern is *Ae. aegypti*, due to its high vector competence for multiple VBDs, its adaptation to urban environments, its previous presence in Europe, and a favourable climate for its establishment in many areas, particularly in the Mediterranean.^5^^,^^7^^,^^72^ Furthermore, there is a lack of understanding regarding the behaviour of *Ae. aegypti* under Mediterranean conditions and a dearth of reliable and formal guidelines and tools for monitoring and control in a European context.

Cyprus is an island in the eastern Mediterranean Sea, divided in 6 administrative districts with a highly suitable climate for many mosquito species. In November 2021, *Ae. aegypti* was detected in the Republic of Cyprus for the first time since 1959, after being introduced in 1934. In 2022, it was confirmed to have overwintered in the coastal district of Larnaca, near the airport and in the same year, *Ae. albopictus* introduction was recorded in Limassol.^95^ Following the confirmation of the re-introduction of *Ae. aegypti* and the introduction of *Ae. albopictus* in Cyprus, the International Atomic Energy Agency (IAEA) coordinated several expert missions to assess the situation and provide advice accordingly. The IAEA team developed an Emergency Response Contingency Plan and submitted it to the Republic of Cyprus in April 2023, with the objective of eradicating *Ae. aegypti* and suppressing *Ae. albopictus*.

The recommendations in the plan were adopted by the country's Ministry of Health (MoH) and established by the Steering Committee for Vectors and Vector-borne Disease, with a range of key stakeholders, including the public health services who are responsible for implementing most of the interventions. Challenges arose from a lack of guidelines and tools and insufficient knowledge and resources. To meet some of the shortcomings, staff had to be hired, relocated or retrained. Despite these challenges, the MoH established standard operating procedures and action plans for future events including the management of imported *Aedes*-borne diseases. The interventions implemented by the multi-sectoral response team are highlighted below:

1. Clinical case and risk management–Clinicians were promptly notified about the potential presence of patients with VBDs transmitted by *Aedes* mosquitoes, including guidance on identifying clinical signs and managing these cases effectively. Reference laboratories were equipped and trained for testing purposes. Additionally, serological surveillance for WNV was already established through blood bank testing during the mosquito season, allowing for adjustments to monitor other VBDs if needed.
2. Vector management–A National Surveillance Network was established to monitor the situation, including the country's entry points, via a combination of trapping techniques. Control measures included the use of adulticides, larvicides and door-to-door activities in properties and public areas such as parks, cemeteries and drains. In collaboration with IAEA, an SIT control trial effort was conducted demonstrating a significant reduction in both the eggs and the adult mosquito populations.
3. Adapting the built environment–Door-to-door activities in and around the infested areas led to the implementation of suppression efforts and interventions on private properties via infrastructure modification to remove and reduce vector breeding sites in combination with chemical control where needed. However, there have been challenges in trying to persuade individuals to alter or adapt their living conditions for vector control.
4. Data harmonisation and decision-support tools–The data generated through entomological surveillance are crucial for mosquito control programmes, particularly for invasive mosquito species in new areas or at points of entry, while early warning systems are critical to detect new introductions. However, significant differences continue globally regarding the types of traps used and the duration of sampling events.^45^^,^^46^ This lack of standardization underscores the need for harmonization to develop common decision-support tools, particularly those which can be shared across borders.
5. Community engagement–Widespread communication and awareness-raising strategies have been implemented, particularly during the typical mosquito season. The educational campaign aimed at raising awareness for the eradication of breeding sites in private areas to eliminate *Aedes* mosquitoes. Additionally, these strategies along with door-to-door activities are enhancing education and participation while improving the acceptance of proper biocide use and the implementation of SIT. An additional elementary school campaign is currently under development.

By targeting both *Ae. aegypti* and *Ae. albopictus*, the cost-effective approach taken in the Republic of Cyprus is expected to reduce the risk of arboviral transmission via both of these invasive mosquito species.^11^ The challenges in terms of training and human resources show an even greater need for cross-border sharing of resources and knowledge, to prevent onward expansion, which will be far more likely if *Ae. aegypti* is introduced to mainland Europe. The IAEA and the European Commission continue to support the Republic of Cyprus in establishing a small-scale mass production factory for sterile mosquitoes in Larnaca and continued commitment from the EU and its member states will be crucial in limiting the spread of *Ae. aegypti.*

## Invention toolkit to resist emerging VBD in Europe

To strengthen resilience to emerging VBD threats, Europe can learn from endemic regions at multiple levels (i.e., community, municipal, national and regional), by adopting advances in technology, infrastructure, clinical practices, and innovative solutions. Resources and financing are often limited, with urgent and shifting priorities at all levels, from governments and NGOs to scientists, medical professionals and the public. Currently, promising areas for immediate scale-up include improved tracking of emerging infectious diseases, from introduction to outbreaks, and expanded asymptomatic and serological data surveillance. More sustained cross-border collaboration and harmonisation of data and management plans are needed moving forward. For example, the establishment of European expertise networks linked to endemic countries to develop optimal clinical care strategies and timely response and support during outbreaks or isolated cases. However, we recognise the challenges with person-to-person networking including financial, logistical and time constraints. Additionally, there is a loss of individual expertise in science and policy as professionals move to other fields and a systemic lack of indigenous and local knowledge, much of which has not been incorporated and integrated into established structures. Continued learning is essential for refining targeted and appropriate VBD control and management strategies at different levels and demonstrating to policymakers the necessity to act proactively against these emerging threats. Table 2 summarises key interventions discussed throughout this Personal View, and potential challenges to achieving these goals in the future in Europe.

**Table 2:** An emerging VBD Intervention Toolkit, sumarising key interventions outlined in this Personal View, and challenges to their implementation in Europe.

Theme	Intervention	Challenges
Clinical case & risk management	➢ Harmonised plan for importation events, including a clear chain of events and clinical symptoms, to map the full epidemiological history of the pathogen in a clinical context ➢ Publishing epidemiological histories in bulletins ➢ Centralised system for reporting ➢ Expand testing capabilities ➢ Emerging VBD education and training for healthcare professionals ➢ Ensure adequate isolation facilities to reduce nosocomial transmission and ensure the protection of staff and other patients ➢ Regional and international networks for clinical management expertise, to ensure rapid access to global experts and clinical leaders from endemic areas	➢ Fragmentation of best practices and mandates ➢ Privatised healthcare ➢ Cuts to healthcare budgets ➢ Time constraints and a lack of trained healthcare professionals globally ➢ Different resources across countries and centres ➢ Long-term continuity challenges due to funding and coordination
Vector management	➢ Consider all entry points of humans, animals and vectors as potential hotspots e.g., airports, seaports, car border crossing and bus and train terminals ➢ Surveillance of the various transmission pathways (humans, animals, vectors and the environment), to better understand signals of outbreak initiation and key risk factors ➢ Widespread surveillance, leading to targeted interventions to improve cost-effectiveness and resource allocation ➢ Continued innovation in novel methods for controlling and monitoring vectors such as classical SIT and smart traps ➢ All traditional and new surveillance/control methods should be organised and integrated to optimise the available tools ➢ Sharing facilities and resources with neighbouring countries	➢ Surveillance fragmentation and coordination issues, especially at the local level ➢ Movement of humans, animals, plants and goods ➢ Legal challenges and poor acceptance in implementing different control strategies ➢ Insufficient funding ➢ Rising insecticide resistance and a lack of new insecticides being developed ➢ Nature-based solutions e.g., water beetles and larvivorous fish, are yet to be fully understood and have received too little attention so far
Adapting the built environment	➢ Legal enforcement of infrastructure modifications and/or vector breeding site removal on private properties ➢ Ensure interventions are tailored to local architecture, customs and behaviours ➢ Test interventions on a smaller trial scale before making larger financial investment ➢ Design and implement VBD interventions to tackle multiple species	➢ Differing laws surrounding the implementation of VBD control on private properties ➢ Potential for new vectors to become adapted to the urban environment ➢ Infrastructure budgets and changing of political leadership and priorities
Data harmonisation and early warning systems	➢ Co-creation of EWS, incorporating the knowledge of scientists, health professionals and policy makers ➢ Build an epidemiological history for each disease, including imported cases and autochthonous cases, and encourage surveillance of the vector and hosts including serology to build “baseline” databases ➢ Better harmonisation, availability and accessibility of data for multiple actors at sufficient temporal and spatial scales ➢ Syndromic surveillance to improve alert timeliness	➢ Data protection and ethics ➢ Data fragmentation ➢ Hacking and security issues of data stores, platforms and applications ➢ Lack of mandatory reporting for important non-notifiable diseases, e.g., leishmaniasis
Community engagement	➢ Community engagement, with support from relevant professionals at strategic times, appears key to scale the implementation of vector control strategies and for effectively managing clinical risks ➢ Identify communities and individuals most at need ➢ Education campaigns cannot solely rely on information dissemination and need direct involvement from scientific staff and community leaders ➢ Target a range of ages, genders, socio-economic groups, races, ethnicities and religions ➢ Enhancing community participation ensures better acceptance and uptake of VBD control	➢ VBDs are not a priority in certain countries, including many in Europe ➢ People can be very resistant to change their behaviours ➢ Community sampling for engagement activities can be selected randomly, without ensuring balanced representation of different groups including socio-economic status, educational attainment and gender, increasing bias

## Conclusion

By integrating diverse perspectives from various geographic regions, disciplines, and areas of expertise, we have developed an Intervention Toolkit that provides a range of approaches for preparedness and control strategies targeting emerging vector-borne diseases (VBDs) in Europe. This toolkit highlights the need for collaborative partnerships between emerging and endemic regions. However, implementing interventions across different geographic areas is often challenging due to differences in culture, economics, climate, nature and biodiversity, laws and other risk factors. In this work we provide examples of cross-country knowledge-sharing efforts and identify potential logistical barriers that may arise. Despite these challenges, many of the partnerships and interventions discussed here have demonstrated progress in vector and disease control and prevention and while the interventions included represent the expertise of our research group, many others exist and are acting synergistically to reach this common goal. We hope that showcasing these collaborative efforts will encourage more knowledge-sharing partnerships between VBD-endemic and -emerging countries, reducing the burden of VBDs not only in Europe but globally.

## Contributors

GECC, TA and RL conceived the study. All authors provided input and expertise into the layout, content and key messages of the Viewpoint. GECC drafted the initial version and incorporated feedback on multiple revisions. All authors contributed input to multiple revisions and have approved the final version. GECC and RL assessed and validated the data and sources used in this Personal View and were responsible for the decision to submit the manuscript.

## Data sharing statement

Data and code used in the creation of the Figures generated for this Personal View are available at: https://earth.bsc.es/gitlab/ghr/vbd-control-europe.

## Declaration of interests

RBM is an employee of Rentokil, a private entity involved in vector control. However, Rentokil had no role in this article and the views expressed here are solely those of the authors and do not necessarily reflect those of any entity or institution with which they are associated. All other authors have no competing interests.

## Appendix A Supplementary data

Supplementary Figures and Table

Abstract portugues

Abstract french

Abstract espanol
