# Supplementary material for: Strengthening resilience to emerging vector-borne diseases in Europe: lessons learnt from countries facing endemic transmission
Source: Lancet Reg Health Eur. 2025 Apr 4;53:101271. doi: 10.1016/j.lanepe.2025.101271 (PMC12002787; doi:10.1016/j.lanepe.2025.101271)
Supplement: Supplementary Figures and Table [file mmc1.pdf]

Supplementary Material

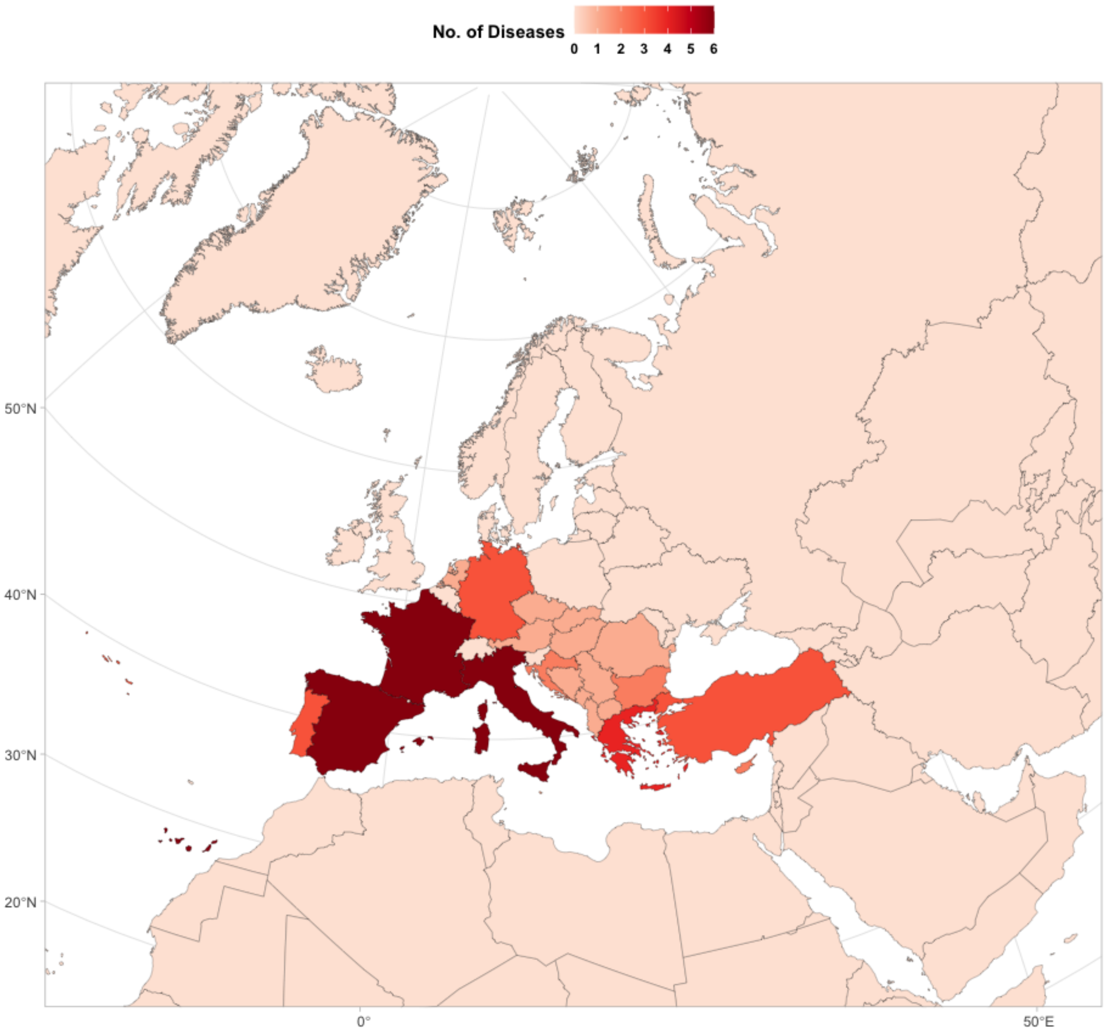

Figure S1: Reported autochthonous transmission for each VBD of concern in Europe (malaria, cutaneous leishmaniasis, Crimean-Congo Haemorrhagic Fever, West Nile virus, chikungunya, dengue, Zika and Oropouche virus) by country, as of 2024. Source: European Centre for Disease Prevention and Control.

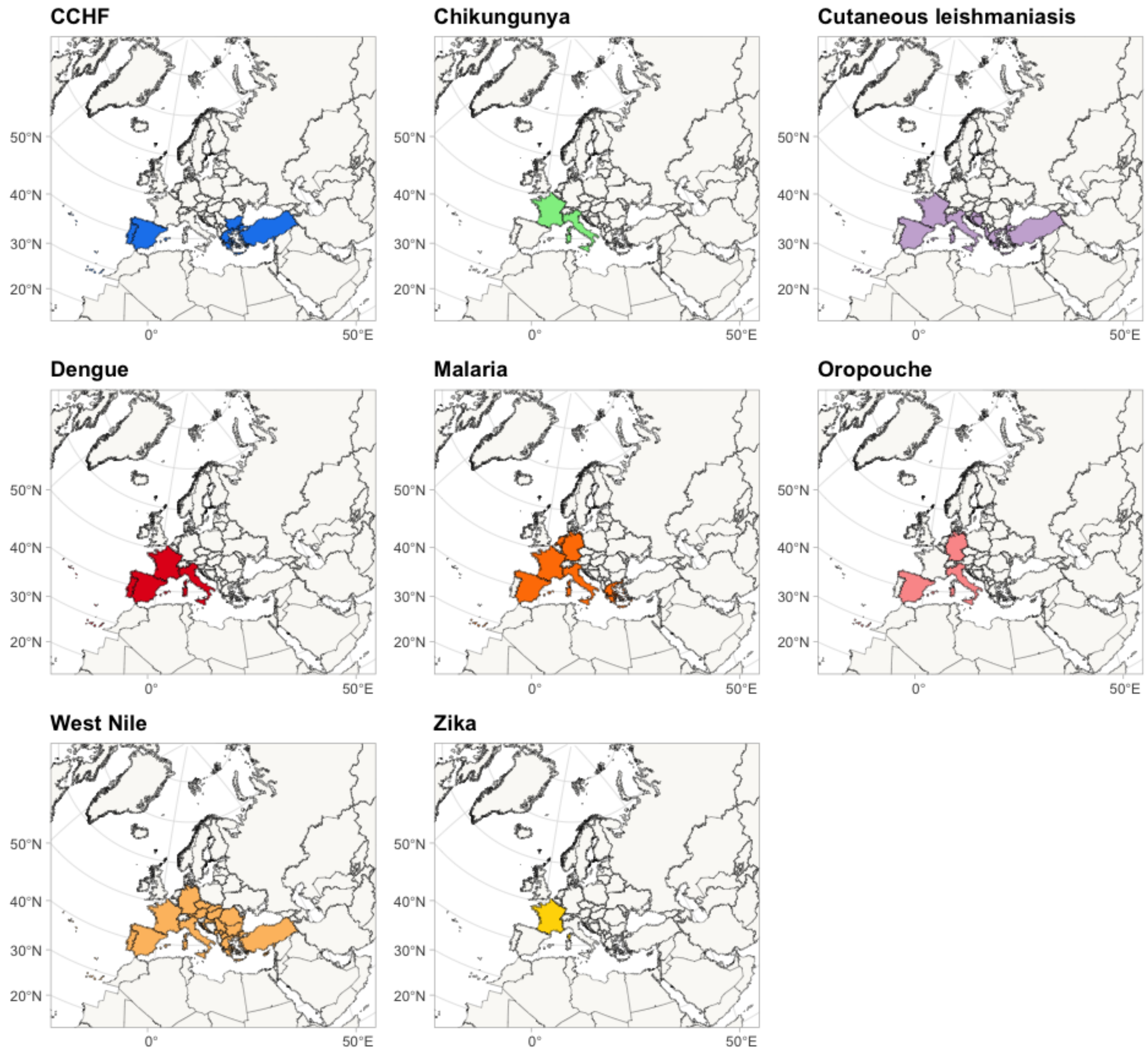

Figure S2: Reported autochthonous transmission (travel-related only for Oropouche) for each VBD, by country and disease, as of 2024. CCHF: Crimean-Congo Haemorrhagic Fever. Source: European Centre for Disease Prevention and Control (due to gaps in reporting, these maps may not represent the true distribution).

Table S1: Countries (ISO2) included in the definition of Europe used here, the European Environment Agency (EEA) member and cooperating countries, plus the United Kingdom. Countries in the European Union are in blue. * Designation is without prejudice to position on status and is in line with UNSCR 1244 and the ICJ Opinion on the Kosovo Declaration of independence.

**Member countries**			
Austria (AU)	Belgium (BE)	Bulgaria (BG)	Croatia (HR)
Cyprus (CY)	Czechia (CZ)	Denmark (DK)	Estonia (EE)
Finland (FI)	France (FR)	Germany (DE)	Greece (GR)
Hungary (HU)	Iceland (IS)	Ireland (IE)	Italy (IT)
Latvia (LV)	Liechtenstein (LI)	Lithuania (LT)	Luxembourg (LU)
Malta (MT)	Netherlands (NE)	Norway (NO)	Poland (PO)
Portugal (PT)	Romania (RO)	Slovakia (SK)	Slovenia (SI)
Spain (ES)	Sweden (SE)	Switzerland (CH)	Türkiye (TR)
**Cooperating countries**			
Albania (AL)	Bosnia and Herzegovina (BA)	Kosovo* (XK)	Montenegro (ME)
North Macedonia (MK)	Serbia (RS)		
**Other countries**			
United Kingdom (GB)			

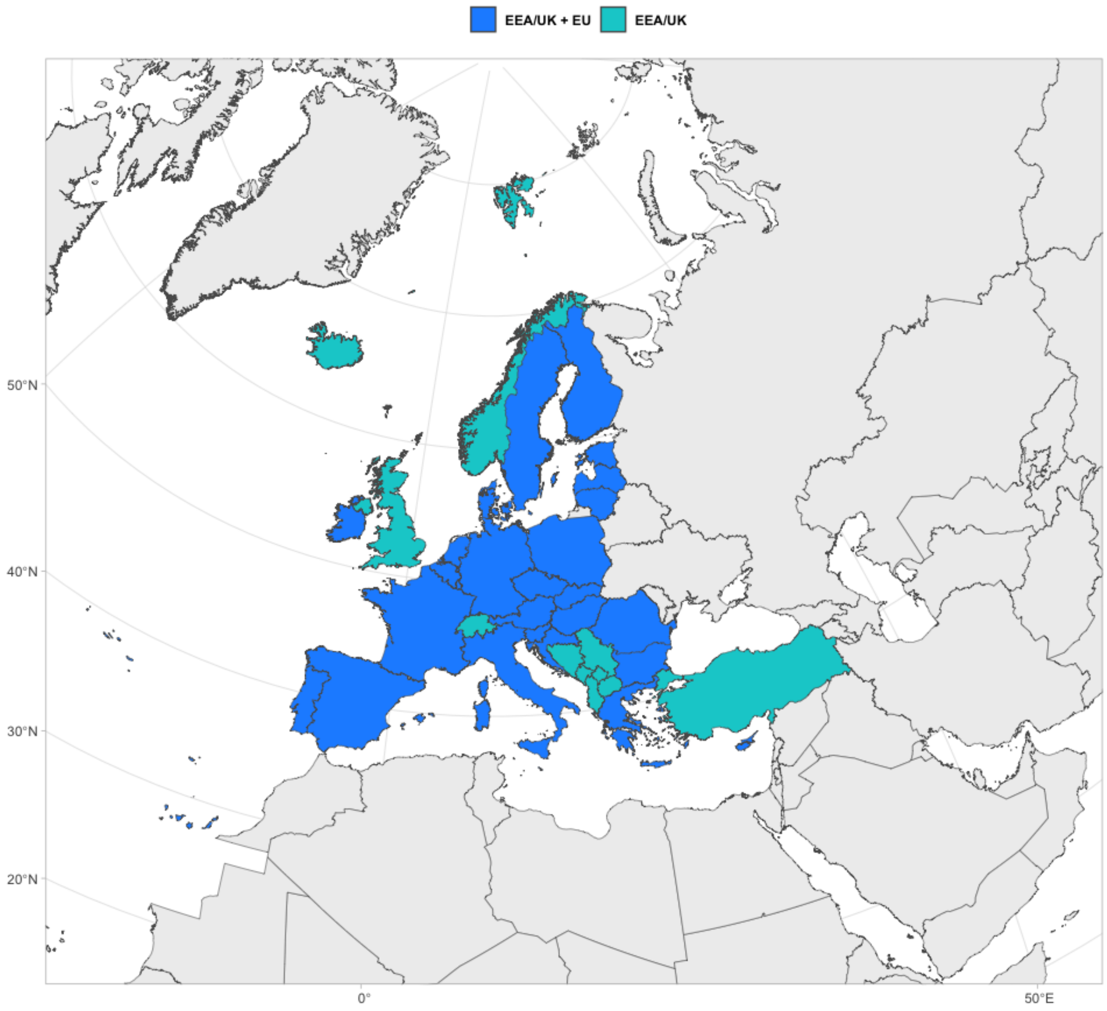

Figure S3 European Environment Agency (EEA) member and cooperating countries and the United Kingdom (UK). Countries within the European Union (EU) are shaded in blue (see Supplementary Material). In this Viewpoint, we consider this grouping of European countries.
