# Supplementary material for: Strengthening resilience to emerging vector-borne diseases in Europe: lessons learnt from countries facing endemic transmission
Source: Lancet Reg Health Eur. 2025 Apr 4;53:101271. doi: 10.1016/j.lanepe.2025.101271 (PMC12002787; doi:10.1016/j.lanepe.2025.101271)
Supplement: Abstract portugues [file mmc2.docx]

*This translation in Portuguese was submitted by the authors and we reproduce it as supplied. It has not been peer reviewed. Our editorial processes have only been applied to the original abstract in English, which should serve as reference for this manuscript.*

Transmitidas por vetores (VBDs) são uma grande preocupação de saúde pública em todo o mundo. As mudanças climáticas, a degradação ambiental e a globalização têm levado a uma expansão da distribuição de muitos vetores e à erosão das barreiras de transmissão, aumentando a exposição humana a novos patógenos e o risco de surtos emergentes de VBD. A Europa está potencialmente despreparada para a crescente ameaça das VBDs, pois a atenção e os recursos têm sido direcionados a outras prioridades de saúde pública. Abordagens de prevenção proativas, em vez de reativas, podem reduzir substancialmente o custo socioeconômico das VBDs. Globalmente, os países endêmicos possuem décadas de experiência no controle das VBDs, e a Europa tem muito a aprender com esse conhecimento. Aqui, defendemos a expansão do compartilhamento de conhecimento transdisciplinar para cocriar medidas proativas contra as VBDs. Apresentamos as experiências e a expertise da nossa diversificada equipe internacional e exploramos como uma variedade de intervenções pode ser aplicada e adaptada para o contexto europeu.
