# Supplementary material for: Strengthening resilience to emerging vector-borne diseases in Europe: lessons learnt from countries facing endemic transmission
Source: Lancet Reg Health Eur. 2025 Apr 4;53:101271. doi: 10.1016/j.lanepe.2025.101271 (PMC12002787; doi:10.1016/j.lanepe.2025.101271)
Supplement: Abstract french [file mmc3.docx]

*This translation in French was submitted by the authors and we reproduce it as supplied. It has not been peer reviewed. Our editorial processes have only been applied to the original abstract in English, which should serve as reference for this manuscript.*

Les maladies à transmission vectorielle (MTV) émergentes constituent une préoccupation majeure en matière de santé publique à l’échelle mondiale. Le changement climatique, la dégradation de l’environnement et la mondialisation ont entraîné une expansion de l’aire de répartition de nombreux vecteurs et une érosion des barrières de transmission, augmentant ainsi l’exposition humaine à de nouveaux agents pathogènes et le risque d’épidémies de MTV émergentes. L’Europe est potentiellement sous-préparée face à cette menace croissante, en raison de l’attention et des financements détournés vers d’autres priorités de santé publique. Une approche de prévention et de contrôle proactive, plutôt que réactive, pourrait considérablement réduire l’impact socio-économique des MTV. Les pays endémiques à travers le monde disposent de décennies d’expérience dans la lutte contre ces maladies, et l’Europe a beaucoup à apprendre de ces connaissances. Nous plaidons ici pour l’expansion de partenariats transdisciplinaires de partage des savoirs, afin de co-créer des mesures proactives contre les MTV. Nous présentons l’expérience et l’expertise de notre équipe internationale diversifiée et explorons comment un éventail d’interventions peut être appliqué et adapté au contexte européen.
