# Supplementary material for: Strengthening resilience to emerging vector-borne diseases in Europe: lessons learnt from countries facing endemic transmission
Source: Lancet Reg Health Eur. 2025 Apr 4;53:101271. doi: 10.1016/j.lanepe.2025.101271 (PMC12002787; doi:10.1016/j.lanepe.2025.101271)
Supplement: Abstract espanol [file mmc4.docx]

*This translation in Spanish was submitted by the authors and we reproduce it as supplied. It has not been peer reviewed. Our editorial processes have only been applied to the original abstract in English, which should serve as reference for this manuscript.*

Las enfermedades emergentes transmitidas por vectores (ETV) son un importante riesgo para salud pública a nivel mundial. El cambio climático, la degradación ambiental y la globalización han provocado una expansión en el rango de muchos vectores y una erosión de las barreras de transmisión, lo que aumenta la exposición humana a nuevos patógenos y el riesgo de brotes de ETV emergentes. Europa podría no estar suficientemente preparada para la creciente amenaza de las ETV, ya que la atención y los fondos se han desviado hacia otras prioridades de salud pública. Los enfoques de prevención y control proactivos, en lugar de reactivos, pueden reducir significativamente el impacto socioeconómico de estas enfermedades. Los países endémicos en todo el mundo tienen décadas de experiencia en el control de las ETV, y Europa tiene mucho que aprender de este conocimiento. Por esto, abogamos Personal View por un intercambio fluido de conocimiento entre estos, con el fin de crear conjuntamente medidas proactivas contra las ETV. Presentamos diversas experiencias enriquecedoras de nuestro diverso equipo internacional y exploramos cómo se pueden aplicar y adaptar una variedad de intervenciones al contexto europeo.
